# Inequalities in overweight and obesity among reproductive age group women in India: evidence from National Family Health Survey (2015–16)

**DOI:** 10.1186/s12905-022-01786-y

**Published:** 2022-06-02

**Authors:** Pradeep Kumar, Sherry Mangla, Sampurna Kundu

**Affiliations:** 1 International Institute of Population Sciences, Deonar, Mumbai, 400088 India; 2 Centre of Social Medicine and Community Health, Jawaharlal Nehru University, Delhi, 110067 India

**Keywords:** Overweight, Obesity, Inequalities, Concentration index

## Abstract

**Background:**

In developing nations like India, fertility and mortality have decreased, and diseases related to lifestyle have become more common. Females in India are more prone to being overweight and obese than their male counterparts, more specifically in affluent families than the poor ones. Understanding the overweight and obesity trend may help develop feasible public health interventions to reduce the burden of obesity and associated adverse health outcomes.

**Methods:**

The study utilizes the fourth round of the National Family Health Survey (NFHS-4), 2015–16. Descriptive statistics, bivariate and multivariate analysis was used to check the significant relationship between overweight and obesity, and other background characteristics. Income-related inequality in overweight and obesity among women was quantified by the concentration index and the concentration curve. Further, Wagstaff decomposition analysis was done to decompose the concentration index, into the contributions of each factor to the income-related inequalities.

**Results:**

Overweight & obesity among women had a significant positive association with their age and educational level. The odds of overweight and obesity were 57% more likely among women who ever had any caesarean births than those who did not [AOR: 1.57; CI: 1.53–1.62]. The likelihood of overweight and obesity was 4.31 times more likely among women who belonged to richest [AOR: 5.84; CI: 5.61–6.08] wealth quintile, than those who belonged to poor wealth quintile. Women who ever terminated the pregnancy had 20% higher risk of overweight and obesity than those who did not [AOR: 1.20; CI: 1.17–1.22]. The concentration of overweight and obesity among women was mostly in rich households of all the Indian states and union territories. Among the geographical regions of India, the highest inequality was witnessed in Eastern India (0.41), followed by Central India (0.36).

**Conclusion:**

The study results also reveal a huge proportion of women belonging to the BMI categories of non-normal, which is a concern and can increase the risks of developing non-communicable diseases. Hence, the study concludes and recommends an urgent need of interventions catering to urban women belonging to higher socio-economic status which can reduce the risks of health consequences due to overweight and obesity. Development nutrition-specific as well as sensitive interventions can be done for mobilization of local resources that addresses the multiple issues under which a woman is overweight or obese.

## Introduction

The prevalence of overweight and obesity is swiftly escalating, with around 19 billion overweight and 650 million obese adults worldwide. The proportion of the overweight or obese population has substantially increased over the last three decades [1], especially in the low-income and middle-income countries like Bangladesh and Nepal [2, 3]. The growth of obesity in developed nations, which began in the 1980s and hastened between 1992 and 2002, has slowed down since 2016. Conversely, in developing nations, almost two-third of the world’s obese population is currently living and is expected to rise in the upcoming years [4]. As per the global estimates of 2016, among the 13% obese adults, around 15% of women are obese, whereas men account for 11% of the total world obese population [5]. Under the continued circumstances, it is estimated that by 2025, 18% of the men and 21% of the women worldwide will be obese [6].

The issue of overweight and obesity is vital in public health. Individuals with overweight and obesity bear many non-communicable diseases (NCDs) like diabetes, high blood pressure, some types of cancers, cardiovascular diseases, and muscular-skeletal disorders [7]. Changes in the dietary pattern have shown to be a significant underlying factor for the increasing prevalence of overweight/obesity and associated complications [8]. This problem is even more severe in the case of women as it affects their as well as their progeny’s life. It is because women with overweight or obesity are likely to suffer from several complications related to pregnancy such as gestational diabetes, gestational hypertension, pre-eclampsia and postpartum haemorrhage, preterm birth and perinatal death for the new-born [9–11].

In developing nations like India, high risk of obesity and related consequences are seen over the time. Fertility and mortality have decreased, and diseases related to lifestyle have become more common [12–14]. In India, more than 135 million population was affected by obesity [15]. Females here are more prone to being overweight and obese than their male counterparts, more specifically in affluent families than the poor ones [15, 16]. As per the National Family Health Survey (NFHS-4) findings, 20.7% of women and 18.9% of men are overweight or obese compared to 12.6% of women and 8.9% of men in 2005–06 [17]. This rapid increase has been accompanied by notable growth in the burden of NCDs [12]. The risk for these NCDs worsens with an increase in Body Mass Index (BMI) [18]. More recently, many studies confirmed a direct relationship between BMI and mortality. In a study in 2016, it was found that all-cause mortality was lowest in the 20–25 kg/m^2^ BMI range. Mortality significantly rose just below this range and throughout the overweight/obese range [19].

In order to determine the underlying predictors for overweight and obesity in women of reproductive age, various researches across different regions have been carried out in the past [20–23]. Some of these researches opined that the proportion of overweight and obesity grows with women’s age, education, and parity [16, 24]. The physiological and psychological changes with childbirth also affect women’s food intake habits and nutritional status [25]. Moreover, endometrial implantation and other reproductive functions get impacted in obese women with complications including delayed conceptions, increased miscarriage rate, and reduced outcomes in assisted conception treatments [26]. Maternal obesity is observed to be related to childhood obesity as well [27].

The physical well-being and maintenance of the everyday health of an individual are related closely to the status of nutrition. Age, educational level, level of richness, factors associated with pregnancy and parity, food habits and lifestyle seem to have been the major factors associated with higher prevalence of overweight and obesity among females. However, in the Indian context, there are no study of evidence in literature identifying the socio-economic inequalities in overweight and obesity. Hence, this study focuses on the socio-economic inequalities in India’s increasing overweight and obese female population and identifying the underlying contributing factors. We used nationally representative data to estimate the patterns in overweight and obesity and to identify socio-demographic factors associated with overweight and obesity among women of reproductive age in India. Without knowing the extent of inequality and its underlying contributing factors, would pose difficulty in guiding policies and resource allocations. Hence, the study will help in developing expedient public health interventions for reducing the burden of obesity and associated adverse consequences.

## Materials and methods

### Data

**Fig. 1:**
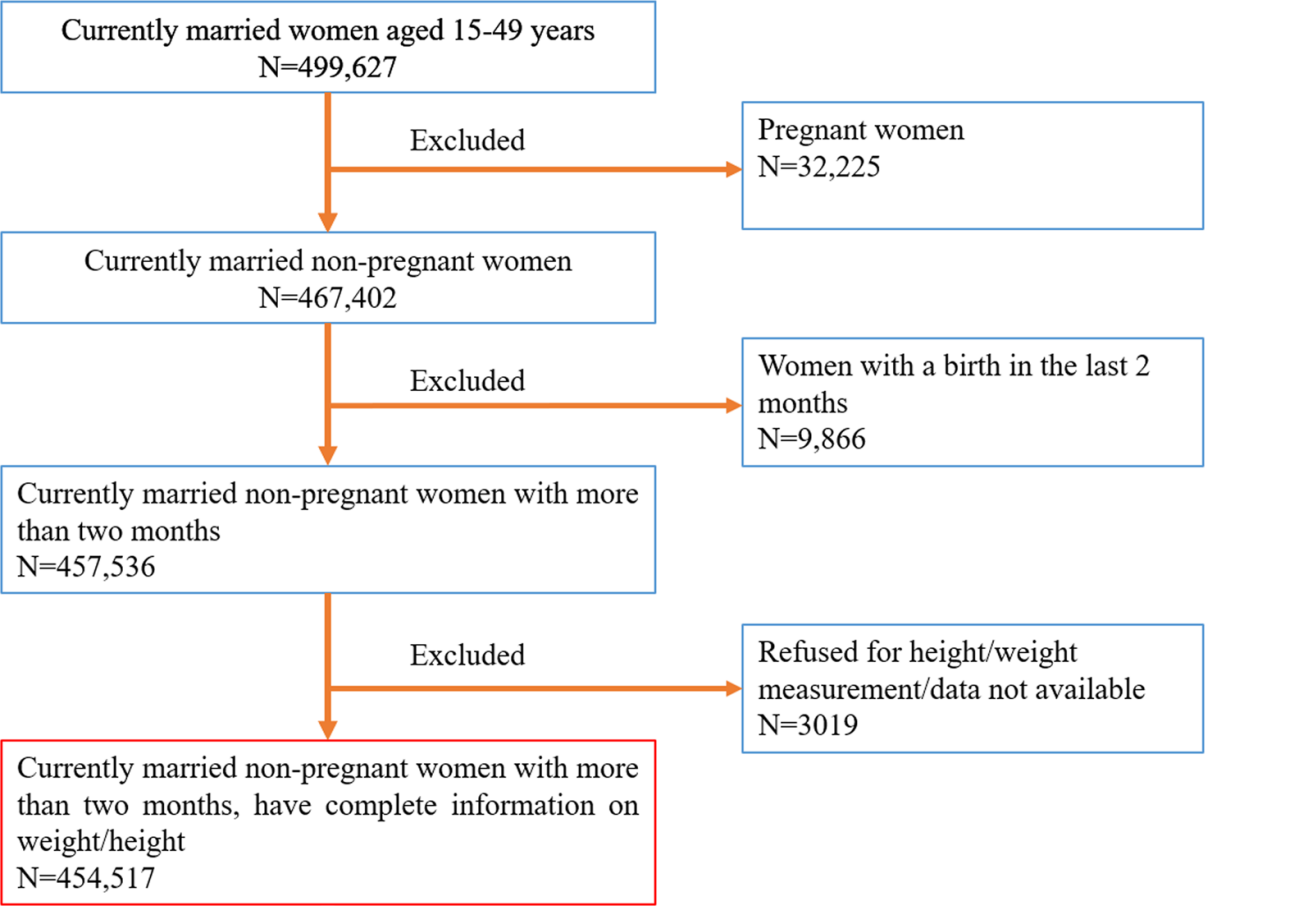
Flowchart showing sample selection

The study utilizes the fourth round of the National Family Health Survey (NFHS-4), a nationally representative cross-sectional survey conducted in 2015–16 under the Ministry of Health and Family Welfare, Government of India. The survey provides detailed information on fertility, family planning, reproductive right and health issue, gender issues, women empowerment, and domestic violence. The survey used a two-stage stratified sampling design to give the estimates at the state and district level, where the sampling frame was taken from the national census for the selection of primary sampling units (PSUs). PSUs were villages in rural areas and Census Enumeration Blocks (CEBs) in urban areas. PSUs with fewer than 40 households were linked to the nearest PSU. Villages were selected from the sampling frame with probability proportional to size (PPS) within each rural stratum. The detailed methodology and complete information on the survey design and data collection published elsewhere [28]. The survey collected information from 601,509 households, 699,686 women, and 112,122 number of men for the response rate of 98 percent, 97 percent, and 92 percent respectively. The study considered only those women who have complete data on the anthropometric outcomes. We have excluded women who were currently pregnant during the survey and women with a birth in the last 2 months as their weight would not be representative. After exclusion, the final sample size for this study was 454,517 (**Fig. ****1**).

We test the hypothesis-

#### H_0_

There exists no economic inequality in overweight and obesity among reproductive age-group women.

Against,

#### H_1_

There exists economic inequality in overweight and obesity among reproductive age-group women.

### Outcome variable

The prevalence of overweight & obesity is the outcome variable of this study. It is challenging to measure overweight and obesity with a single index. However, the Body Mass Index (BMI) is the most widely used index for determining adult overweight and obesity. The cutoff of overweight and obesity was defined as per WHO guidelines[29], i.e., BMI ≥ 25 kg/m^2^. Women having BMI ≥ 25 kg/m^2^ were coded “1” overweight and obesity and “0” otherwise.

### Explanatory variables

The primary exposure variable was household’s wealth index which is often used to measure the household economic status in Demographic and Health Surveys (DHS). Data on asset holdings and other household characteristics were used to create the wealth index. Scores are assigned to households based on the number and types of consumer goods they own, ranging from a television to a bicycle or car and housing characteristics such as water supply, toilet facilities, and flooring materials. Principal component analysis was used to calculate these scores. The national wealth quintiles are calculated by assigning a score to each usual (de jure) household member, ranking each person in the household population according to their score, and dividing the distribution into five equal categories, each with 20% of the population [28]. Additionally, the study included women’s age, educational attainment, caste, religion, place of residence, geographical region, household size, number of children, caesarean section (ever had any caesarean section delivery), termination of pregnancy (ever had a terminated pregnancy), watching TV, and food habit (eats fast food). Women’s age was categorized as 15–19, 20–29, 30–39, and 40–49 years. Educational level was grouped as no education, primary, secondary, and higher. Caste was classified as scheduled caste (SC), scheduled tribe (ST), other backward class (OBC), and others. Religion was categorized as Hindu, Muslim, and others. Place of residence was available in the survey as urban and rural. Geographical regions were divided into six categories as North, Central, East, Northeast, West, and South. Household size was categorized as 1–4, 5–6, and 6 & above. Number of children were defined as 1–2, 3–4, and above 4. If respondents ever had a caesarean section delivery, they were asked to answer 'Yes', and 'No' if they had never had a caesarean section delivery while termination of pregnancy was coded as yes and no. If women watched television at less than once a week/least once a week/almost every day, they were categorized as ‘Yes’, and if they did not, they were categorized as ‘No’. Fast food was constructed with the help of variables “frequency of eats fried food and takes aerated drinks. It was coded as “1” yes if respondent eats/takes either one daily or weekly and “0” no, otherwise.

### Statistical analysis

Descriptive statistics along with bivariate analysis was used to observe the distribution and association of the variables. A chi-square test was performed to test significant association between outcome and other explanatory variables. Additionally, binary logistic regression analysis was used to check the significant relationship between overweight and obesity, and other background characteristics. The adjusted odds ratio with a 95% confidence interval were presented in the results.

### Concentration Index (CCI)

Income-related inequality in overweight and obesity among women was quantified by the concentration index (CCI) and the concentration curve (CC), using the household wealth score (continuous) as the socio-economic indicator and binary outcome as overweight and obesity. The concentration curve is obtained by plotting the cumulative proportion of overweight and obesity against the cumulative proportion of the women ranked by the socio-economic indicator [30, 31]. The concentration index can be written as follows:

$$C = \frac{2}{\mu }cov\left( {y_{i} ,R_{i} } \right)$$

where C is the concentration index; $y_{i}$ is the outcome variable ; ***R*** is the fractional rank of individual ***I*** in the distribution of socio-economic position; *µ*. is the mean of the outcome variable of the sample, and $cov$ denotes the covariance.

If the curve lies above the line of equality, the concentration index takes a negative value, indicating a dispr oportionate concentration of inequality among the poor (pro-rich). Conversely, if the curve lies below the line of equality, the concentration index takes a positive value, indicating a disproportional concentration of inequality among the rich (pro-poor). In the absence of socio-economic-related inequality, the concentration index is zero.

### Decomposition of the concentration index

The study used Wagstaff decomposition analysis to decompose the concentration index. Wagstaff’s decomposition demonstrated that the concentration index could be decomposed into the contributions of each factor to the income-related inequalities [31]. Based on the linear regression relationship between the outcome variable $y_{i}$, the intercept α, the relative contribution of $x_{ki}$. and the residual error $\varepsilon_{i}$.

$$y_{i} = \alpha + \sum \beta_{k} x_{ki} + \varepsilon_{i}$$

where $\varepsilon_{i}$ is an error term, given the relationship between $y_{i}$ and $x_{ki}$, the CI for y (C) can be rewritten as:

$$C = \sum \left( {\frac{{\beta_{k} \overline{x}_{k} }}{\mu }} \right)C_{k} + \frac{GC\varepsilon }{\mu }/\mu$$

where $\mu$ is the mean of $y_{i}$, $\overline{x}_{k}$, is the mean of $x_{k}$, $\beta_{k}$ is the coefficient from a linear regression of outcome variable, $C_{k}$ is the concentration index for $x_{k}$ (defined analogously to C, and GC_ɛ_ is the generalized concentration index for the error term ($\varepsilon_{i}$).

Here C is the outcome of two components: First, the determinants or ‘explained’ factors. The explained factors indicate that the proportion of inequalities in the overweight and obesity is explained by the selected explanatory factors, i.e., x_k_. Second, a residual or ‘unexplained’ factor $\left( {\frac{GC\varepsilon }{\mu }/\mu } \right)$, indicating the inequality in health variables that cannot be explained by selected explanatory factors across various socio-economic groups.

## Results

**Fig. 2:**
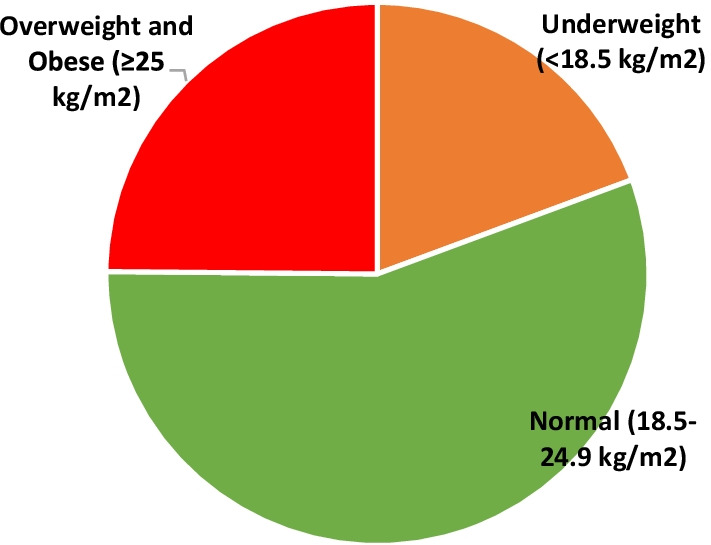
Body mass index (BMI) status of women aged 15–49 years, India, 2015–16

Figure 2 displays the body mass index status of reproductive-age women. About 19% of women were underweight (BMI < 18.5 kg/m^2^), one-fourth were overweight & obese (BMI ≥ 25 kg/m^2^), and the rest of the women had normal BMI (18.5–24.9 kg/m^2^).

### Socio-economic and demographic profile of study population

Table 1 shows that only  3 percent of women belonged to the 15–19 years age group, and a significant proportion of women belonged to age group 20–29 (32.5%) and 30–39 years (36.1%). One-third of women had no education, while only 10 percent of women were higher educated. One-fifth of women belonged to the scheduled caste group. The majority of women were Hindu (81.7%), lived in rural areas (66.2%), and watched TV (76.2%). More than half of the women consumed fast food. Half of the women had a number of children 1–2, and 38 percent of women had household sizes 1–4. About 7 percent of women had ever had any caesarean delivery, and nearly 16 percent had a terminated pregnancy.

**Table 1:** Sample distribution of study population, India, 2015–16

Covariates	Percentage	Sample
Age (in years)		
15–19	2.9	12,234
20–29	32.5	145,662
30–39	36.1	166,035
40–49	28.5	130,586
Education		
No education	33.7	160,836
Primary	14.4	66,824
Secondary	42.0	187,537
Higher	9.8	39,320
Caste		
Scheduled caste	20.1	81,115
Scheduled tribe	9.0	78,177
OBC	43.6	181,237
Others	27.2	113,988
Religion		
Hindu	81.7	346,657
Muslim	12.8	56,724
Others	5.4	51,136
Wealth index		
Poorest	17.7	88,358
Poorer	19.6	97,247
Middle	20.5	94,270
Richer	21.3	89,206
Richest	21.0	85,436
Place of residence		
Urban	33.8	128,248
Rural	66.2	326,269
Region		
North	13.4	91,252
Central	22.1	116,897
East	23.0	85,564
Northeast	3.4	59,831
West	14.6	38,280
South	23.5	62,693
Household size		
1–4	38.1	162,172
5–6	35.4	165,439
Above 6	26.5	126,906
Number of children		
Zero	8.1	35,819
1–2	50.8	214,753
3–4	31.1	150,260
Above 5	10.1	53,685
Ever had any caesarean		
No	93.2	427,861
Yes	6.8	26,656
Ever had a terminated pregnancy		
No	84.1	381,690
Yes	15.9	72,827
Watching TV		
No	23.8	117,848
Yes	76.2	336,669
Eats fast food (daily or weekly)		
No	47.5	219,502
Yes	52.5	235,015
Total	**100.0**	**454,517**

The bold values indicate total percentage contribution of each variable

### Prevalence of overweight and obesity and wealth status

Overweight & obesity among women had a significant positive association with their age and educational level. The odds of overweight & obesity increase with the increased age and education of the women. The prevalence of overweight and obesity was significantly higher among women who belonged to others caste groups (32%) than those who belonged to scheduled caste (21%) and scheduled tribe (12%). Households’ wealth index has a positive association with overweight and obesity among women. The likelihood of overweight and obesity was 4.31 times and 5.84 times more likely among women who belonged to richer [AOR: 4.31; CI: 4.15–4.47] and richest [AOR: 5.84; CI: 5.61–6.08] wealth quintile, respectively than those who belonged to poorest wealth quintile. Women who lived in urban areas had a higher prevalence of overweight and obesity than those in rural areas (38 vs. 18%). Moreover, the odds of overweight and obesity were 57% more likely among women who ever had any caesarean births than those who did not [AOR: 1.57; CI: 1.53–1.62]. Similarly, women who ever terminated the pregnancy had 20% higher risk of overweight and obesity than those who did not [AOR: 1.20; CI: 1.17–1.22]. The likelihood of overweight and obesity was significantly higher among women who watched TV than those who did not [AOR: 1.19; CI: 1.16–1.22]. Women who eat fast food had a higher prevalence of overweight and obesity than those who did not eat (26 vs. 23%) (Table 2).

**Table 2:** Bivariate and logistic regression analysis depicting association between overweight/obese among women aged 15–49 years by their background factors in India, 2015–16

Variables	Overweight & Obesity	*p*-values	AOR (95% CI)
Age (in years)		*p* < 0.0001	
15–19	5.6		Ref
20–29	15.5		2.59***(2.36 − 2.84)
30–39	27.7		5.8***(5.28 − 6.36)
40–49	34.0		8.05***(7.33 − 8.84)
Education		*p* < 0.0001	
No education	17.3		Ref
Primary	23.5		1.18***(1.15 − 1.21)
Secondary	28.9		1.29***(1.27 − 1.32)
Higher	35.4		1.21***(1.17 − 1.25)
Caste		*p* < 0.0001	
Scheduled caste	20.7		0.86***(0.84 − 0.88)
Scheduled tribe	11.8		0.63***(0.62 − 0.65)
OBC	25.3		0.87***(0.85 − 0.88)
Others	31.6		Ref
Religion		*p* < 0.0001	
Hindu	23.7		Ref
Muslim	29.4		1.46***(1.42 − 1.49)
Others	32.5		1.26***(1.22 − 1.29)
Wealth index		*p* < 0.0001	
Poorest	7.0		Ref
Poorer	13.7		1.75***(1.69 − 1.81)
Middle	22.8		2.85***(2.75 − 2.95)
Richer	33.8		4.31***(4.15 − 4.47)
Richest	43.3		5.84***(5.61 − 6.08)
Place of residence		*p* < 0.0001	
Urban	37.8		Ref
Rural	18.3		0.79***(0.78 − 0.81)
Region		*p* < 0.0001	
North	27.1		Ref
Central	19.8		1.00(0.98 − 1.03)
East	18.0		1.07***(1.04 − 1.1)
Northeast	17.1		1.04**(1.02 − 1.07)
West	27.6		0.98(0.96 − 1.01)
South	34.5		1.39***(1.36 − 1.43)
Household size		*p* < 0.0001	
1–4	28.4		Ref
5–6	24.0		0.89***(0.88 − 0.91)
Above 6	20.9		0.85***(0.83 − 0.87)
Number of children		*p* < 0.0001	
0	16.4		Ref
1–2	27.1		1.05***(1.01 − 1.09)
3–4	24.9		1.14***(1.1 − 1.18)
Above 5	20.2		1.16***(1.11 − 1.21)
Ever had any caesarean		*p* < 0.0001	
No	24.4		Ref
Yes	31.1		1.57***(1.53 − 1.62)
Ever had a terminated pregnancy		*p* < 0.0001	
No	24.1		Ref
Yes	28.9		1.20***(1.17 − 1.22)
Watching TV		*p* < 0.0001	
No	12.0		Ref
Yes	28.9		1.19***(1.16 − 1.22)
Eats fast food (daily or weekly)		*p* < 0.0001	
No	23.3		Ref
Yes	26.2		1.03***(1.01 − 1.04)

Ref.: reference category; ***p < 0.0001; **p < 0.05; AOR: Adjusted odds ratio; CI: confidence interval

### Estimates from decomposition analysis for overweight and obesity among women

Table 3 shows the results from decomposition analysis for the contribution of explanatory variables for overweight and obesity among reproductive-age women. The positive scores of concentration index denote that overweight and obesity among women concentrated among rich women for that particular variable and vice-versa. The study found that women aged 20–29 years, primary educated, belonged to scheduled caste/tribe, from North, Central and East region, had household size 5–6 and above 6, with more number of children (3–4 and 4 +) were concentrated amongst the bottom segment of the population in terms of inequalities in overweight and obesity. While women aged older age (30–39 and 40 + years), had advanced education (secondary and higher), urban resident, from South region, ever had a caesarean births, having terminated pregnancy, watching TV, and eats fast food were more concentrated on the upper segment of the population on the question of inequalities of overweight and obesity. Place of residence, women’s education, watching television, geographical region, caste, and women’s age ha d the highest contribution to the inequality in overweight and obesity. Nearly 29% of inequality was explained by place of residence followed by women’s education (22.3%). Watching TV had a significant contribution to overweight and obesity-related inequality (about 15%). About 12% of inequality was explained by region, followed by caste (nearly 8%), and women’s age explained around 8%.

**Table 3:** Estimates of decomposition analysis for contribution of explanatory variables for overweight and obesity among women in India, 2015–16

Variables	Elasticity	Concentration index (CCI)	Absolute contribution	Percentage contribution	
Age (in years)					
15–19					
20–29	0.019	− 0.038	− 0.001	− 1.3	
30–39	0.070	0.011	0.001	1.4	
40–49	0.079	0.054	0.004	7.6	**7.7**
Education					
No education					
Primary	0.007	− 0.134	− 0.001	− 1.8	
Secondary	0.038	0.183	0.007	12.6	
Higher	0.010	0.626	0.006	11.5	**22.3**
Caste					
Scheduled caste	− 0.010	− 0.159	0.002	2.8	
Scheduled tribe	− 0.008	− 0.405	0.003	5.7	
OBC	− 0.013	0.022	0.000	− 0.5	**7.9**
Others					
Religion					
Hindu					
Muslim	− 0.046	− 0.017	0.001	1.4	
Others	0.000	0.008	0.000	0.0	1.4
Place of residence					
Urban	0.038	0.431	0.016	29.1	
Rural					**29.1**
Region					
North					
Central	− 0.005	− 0.150	0.001	1.3	
East	− 0.009	− 0.334	0.003	5.3	
Northeast	− 0.003	− 0.229	0.001	1.1	
West	− 0.002	0.189	0.000	− 0.6	
South	0.012	0.218	0.003	4.5	**11.6**
Household size					
1–4					
5–6	− 0.003	− 0.035	0.000	0.2	
Above 6	− 0.001	− 0.024	0.000	0.1	0.2
Number of children					
Zero					
1–2	0.008	0.139	0.001	1.9	
3–4	0.003	− 0.109	0.000	− 0.7	
Above 5	-0.002	− 0.374	0.001	1.1	2.3
Ever had any caesarean					
No					
Yes	0.005	0.253	0.001	2.0	2.0
Ever had a terminated pregnancy					
No					
Yes	0.005	0.042	0.000	0.4	0.4
Watching TV					
No					
Yes	0.048	0.168	0.008	14.5	**14.5**
Eats fast food (daily or weekly)					
No					
Yes	0.005	0.047	0.000	0.4	0.4
Explained CI		0.06			
Actual CI		0.31			
Residual		0.25			

The bold values indicate total percentage contribution of each variable

**Fig. 3:**
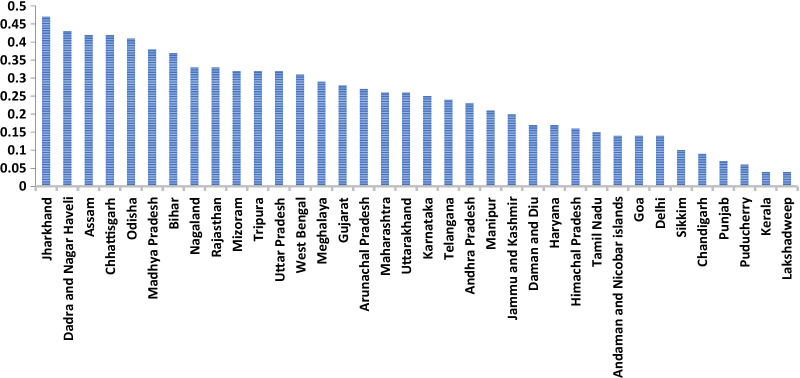
Concentration index for overweight & obesity among women in states/union territories of India

Figure 3 displays the concentration index for overweight and obesity among women in various states of India. Jharkhand (0.47) witnessed the highest inequality for overweight and obesity among women, followed by Dadar and Nagar Haveli (0.43), and Assam (0.42). The higher value of concentration index in Jharkhand signifies that most of the overweight and obesity among women are concentrated in rich household only. Lakshadweep (0.04) witnessed the lowest inequality in overweight and obesity among women, followed by Kerala (0.04), Puducherry (0.06) and Punjab (0.07). The concentration of overweight and obesity among women was mostly in rich households of all the Indian states and union territories.

**Fig. 4:**
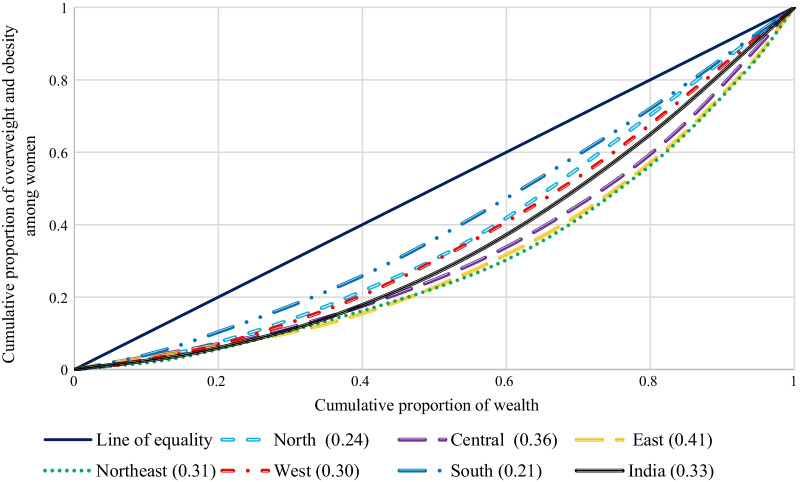
Concentration curve for overweight and obesity among women in India by regions

Figure 4 shows the concentration curve for overweight and obesity among reproductive-age women in India. The concentration curve (CC) lies below the line of equality; it implies that overweight and obesity among women is concentrated among the rich. Moreover, if CC was formed above the line of equality, the inequality would concentrate on the poor and vice-versa. Additionally, the more the areas between the line of equality and curve higher the inequality. India had an inequality of 0.33, which describes that overweight and obesity were concentrated among rich women. Among the geographical regions of India, the highest inequality was witnessed in Eastern India (0.41), followed by Central India (0.36). The lowest inequality was observed in South India (0.21), followed by Northern India (0.24). In all the regions in India, overweight and obesity are concentrated among rich women only.

## Discussion

The present study has thrown light into the current scenario of the burden of overweight and obesity in Indian context and its associated socio-economic and demographic correlates. In India, obesity and overweight has been on rise over the years and has surpassed the global average [32]. Around 25% of the women population in India in the age group 15–49 are overweight or obese, which has increased from the past survey estimates by almost two times. Although the obesity or overweight has been a problem of the developed countries but the epidemic has also started to pose challenges on the developing countries as well. In a developing country like India where double burden of malnutrition and hunger persists, the increasing burden of overweight or obesity may be questionable [33]. But studies and scholars have explained this surging rise due to the transitional phase of nutrition in developing countries that is the shift from traditional Indian diets to adapting Western diets, which includes a combination of sedentary lifestyle, fast food consumption, increased stress, polluted environment and urbanisation [34–36].

The prevalence of overweight or obesity are observed to be increasing with age, indicating positive association between them. Advancement in age comes with the existence of non-communicable diseases [37] and also increasing parity [38] which are common factors leading to overweight or obesity. A study by [39], which had closely studied the association between parity and obesity, reported that multi-parous women had gained weight more due to increase in food intake and/or no physical activities. The women who have higher number of children, caesarean delivery and had a terminated pregnancy are found to have higher chances of being overweight or obese. The pregnancy weight gain that often sustains a lifetime is also an attributable cause and also an highly associated factor with caesarean delivery [27, 40–42].

Among higher educated women, the prevalence of overweight or obesity is higher as evident from the similar relationship from other studies as well [2, 43–45]. Among the other socio-economic variables such as urban residence, higher wealth index and higher social status had positive associations with being overweight or obesity. With higher levels of education come better livelihood opportunities for women that ultimately lead to self-dependency and higher socio-economic status [16]. The study also shows that the overweight or obesity among women is mostly concentrated among the rich. Mostly the employment is of less labour intensive nature involving a sedentary lifestyle with work stress [24, 46, 47]. More purchasing ability allows for getting everything at doorstep without much movement and also unhealthy dietary patterns which is reflected from the study result as well where the likelihood of becoming overweight or obese is high among those who eat more fast foods [48, 49]. All of these factors lead to gain in body weight and ultimately becoming overweight or obese. The differences in the socio-economic and demographic correlates are attributable to the differences in the categories of body weights in various population subgroups. To exemplify, people belonging to the Hindu religion in India are mostly vegetarians who consumes less calorie food as compared to people of other religions life Muslims or Christians [50, 51].

As observed from the regional variations on the prevalence of overweight and obesity in India, the women belonging to the southern states are more overweight or obese in comparison to other states. Plausible reasons could be that the socio-economic status in South Indian states are much higher as female literacy rates are the higher [52, 53]. Furthermore, women living below poverty line are much less in proportion in the Southern states in comparison other states [54]. In India, southern states are much ahead in terms of women empowerment and they enjoy a much better life with good education and income which again brings in the factor of sedentarism and unhealthy lifestyle practices [16]. The framework by [55] had given that under and over-nutrition (in our case overweight/obesity), can be categorized into three levels, that includes- immediate (health and biological factors); underlying (social and environmental factors); and, basic (economic and political). The interaction among these factors result in the nutrition level to be over or under. The increasing urbanization along with better income has led to more access to high calorie food intake, processed food and lack of physical activity, which causes obesity and related diseases [8, 34]. Thus the determinants and correlates which were also identified by other low-to-middle-income countries, confirms the reliability of the study results.

The findings of the present study have significant public health implications by providing empirical evidences on scenario of overweight or obesity among women in India. In a country which comprises of more than one-twelfth of the global women population, and with half of them being in their reproductive age-groups, the results of the study give sufficient representation of national scenario. Women are at elevated risks of developing complications due to high BMIs resulting in becoming overweight or obese, mostly due to the identified lifestyle factors. It is necessary to increase awareness and educate them on the importance of maintaining a healthy weight which will stem from practicing healthy lifestyle. Indian policies that focuses on the marginal and vulnerable populations of the societies that are pro-rural, often ignore the issues of overweight and obesity, which are mostly a problem in the urban areas. There is a need to focus on the growing epidemic of overweight/obesity among urban women, which will further prevent the burden of chronic conditions like diabetes, cardiovascular diseases, hypertension and infertility in India. The urban health programmes can promote healthy diet and physical exercises by incorporating them into special clauses.

The study has some limitations such as, the data being cross-sectional, there is a lack of evidence about temporal relationship due to which some observed factors might not be causally associated. The survey has limited information on the lifestyle habits that includes physical activity and sleep patterns. Though the study captures the socio-demographic, economic, lifestyle and pregnancy factors that determine overweight and obesity, a more detailed study on subjects based on these correlates can help in gaining better understanding.

## Conclusion

The present study depicts the rising levels of overweight or obesity among women in India. Among the important determinants, like increasing age, higher education, urban residence, higher economic status, are the important factors contributing to the prevalence of overweight or obesity among women in the reproductive age groups. The study results also reveal a huge proportion of women belonging to the BMI categories of non-normal, which is a concern and can increase the risks of developing non-communicable diseases. Hence, the study concludes and recommends an urgent need of interventions catering to urban women belonging to higher socio-economic status which can reduce the risks of health consequences due to overweight and obesity. Development of nutrition specific as well as sensitive interventions can be done for mobilization of local resources that addresses the multiple issues under which woman is overweight or obese. Also, an urgent need for focus on the higher wealth quintile women is suggested so that the burden of overweight and obesity can be reduced among them.

## Data Availability

Data was requested and obtained from concerned authorities. The data is freely available in the DHS website on request (https://dhsprogram.com/data/available-datasets.cfm).
